# Evaluating the effects of brain injury, disease and tasks on cognitive fatigue

**DOI:** 10.1038/s41598-023-46918-y

**Published:** 2023-11-17

**Authors:** Glenn R. Wylie, Helen M. Genova, Bing Yao, Nancy Chiaravalloti, Cristina A. F. Román, Brian M. Sandroff, John DeLuca

**Affiliations:** 1 Rocco Ortenzio Neuroimaging Center, Kessler Foundation, 1199 Pleasant Valley Way, West Orange, NJ 07052 USA; 2 Department of Physical Medicine and Rehabilitation, Rutgers University, New Jersey Medical School, Newark, USA; 3 Department of Veterans’ Affairs, The War Related Illness and Injury Center, East Orange Campus, East Orange, NJ 07018 USA; 4 Department of Neurology, Rutgers University, New Jersey Medical School, Newark, NJ 07101 USA

**Keywords:** Predictive markers, Functional magnetic resonance imaging, Magnetic resonance imaging, Striatum

## Abstract

Because cognitive fatigue (CF) is common and debilitating following brain injury or disease we investigated the relationships among CF, behavioral performance, and cerebral activation within and across populations by combining the data from two cross-sectional studies. Individuals with multiple sclerosis (MS) were included to model CF resulting from neurological disease; individuals who had sustained a traumatic brain injury (TBI) were included to model CF resulting from neurological insult; both groups were compared with a control group (Controls). CF was induced while neuroimaging data was acquired using two different tasks. CF significantly differed between the groups, with the clinical groups reporting more CF than Controls—a difference that was statistically significant for the TBI group and trended towards significance for the MS group. The accrual of CF did not differ across the three groups; and CF ratings were consistent across tasks. Increasing CF was associated with longer response time for all groups. The brain activation in the caudate nucleus and the thalamus was consistently correlated with CF in all three groups, while more dorsally in the caudate, activation differed across the groups. These results suggest the caudate and thalamus to be central to CF while more dorsal aspects of the caudate may be sensitive to damage associated with particular types of insult.

## Introduction

Fatigue is an everyday occurrence, yet remains poorly understood. Furthermore, while transient fatigue is ubiquitous in the general population, fatigue is pervasive, persistent, and debilitating for clinical groups such as individuals with multiple sclerosis (MS) and traumatic brain injury (TBI). Indeed, individuals who have sustained a TBI rate fatigue as their worst symptom^1^, contributing to a lower quality of life^2^. Similarly, individuals with MS rate fatigue as amongst their most debilitating symptoms^3^, interfering with activities of daily living and resulting in a lower quality of life^4^. Moreover, in both MS and TBI populations, fatigue has been linked with cognitive impairment^5–8^.

While the importance of understanding fatigue in neurological populations has long been appreciated^2,3^, fundamental knowledge gaps remain. For example, it is unclear if fatigue experienced by individuals with neurological disorders is different from fatigue experienced by healthy individuals (Controls), and if different populations experience different ‘baseline’ levels of fatigue and/or different accrual rates of fatigue. Moreover, the relationships among fatigue, behavioral performance, and activation in associated brain regions within and across populations are poorly characterized. Addressing these issues is critical to develop a clear understanding of what fatigue is and to understand its role in cognition, both of which are necessary to develop effective treatments.

Beyond the knowledge gaps pointed out above, fatigue-related research has been hampered by the fact that “fatigue” is an umbrella term, covering several domains. Here we focus on cognitive fatigue (CF), or fatigue resulting from cognitive work. Additionally, we primarily focus on state CF, or CF that accrues over a relatively short timeframe (e.g., the past four minutes). Trait CF measures, by contrast, focus on CF reported to have accrued over a longer timeframe (e.g., the past four weeks). While trait CF measures may provide a good index of baseline CF levels, they may also be contaminated by other factors (e.g., depression, medication and deconditioning). Indeed, the correlation between trait CF measures and depression is well docuemented^39–42^. In contrast, similar associations between state CF and depression have not been found^9^. Recent research using state CF measures and fatigue-induction paradigms has shown the caudate nucleus of the basal ganglia and the thalamus to play central roles in the experience of fatigue^10–20^.

Here, we combined two datasets^15,21^ in which state CF was induced in individuals with and without neurological damage, focusing specifically on CF. Individuals who had sustained a TBI were included as representative of neurological insult and individuals with MS were included as representative of neurological disease. We used two different fatigue-induction tasks while fMRI data were acquired. This allowed us to compare CF reported by individuals in each of the three groups as well as the rate at which CF increased across tasks. We also assessed the relationship between CF and behavioral performance, the relationship between CF and brain atrophy, and the relationship between CF and brain activation in the three groups. For the purposes of this study, the Blood Oxygen Level Dependent (BOLD) contrast was taken as a proxy for brain activation. Moreover, as CF was induced using two tasks, we were able to assess the stability of CF ratings over time and determine whether the CF experienced was task specific or domain general.

We hypothesized that the two clinical groups would report comparable, high levels of CF relative to the control group (i.e., ‘pathological’ CF). Based in previous research, we hypothesized that CF would increase at approximately the same rate across the three groups^15,21^, and we hypothesized that more CF would be related to longer RTs^22^. Finally, because previous research has shown the thalamus^10,11^ and the caudate of the basal ganglia^10,11,15,16,23,24^ to play important roles in CF, we hypothesized that activation in the thalamus and the caudate would be related to CF in the three groups.

## Methods and materials

### Subjects

This study was a secondary analysis of two datasets and included 30 control participants, 31 individuals with MS, and 31 individuals with TBI for a total of 92 subjects (see Table 1). Because we had access to the original data, we were able to include participants who did not meet the matching requirements of the original studies, despite having valid data. All participants were native English speakers between 20 and 65 years of age, right-handed, and had normal or corrected-to-normal visual acuity. Participants further had no history of drug or alcohol abuse, clinically significant psychiatric history (e.g., schizophrenia or bipolar disorder, major depressive disorder), MRI contraindications, or psychoactive medication use. Participants were excluded if they had any significant neurological history (e.g., head injury, stroke, seizures) with the exception of MS and TBI for the respective groups. While the two original studies included criteria for matching each clinical group to the control group, it was not practical to match all three groups across all demographic variables (e.g., MS is more prevalent in women while TBI is more prevalent amongst men^25,26^). Additionally, because depression is more common in MS^27,28^ and TBI^27,28^ relative to Controls and because fatigue has been shown to covary with depression^29,30^, subjects completed the Chicago Multiscale Depression Inventory^31,32^. All procedures were approved by the Institutional Review Boards of Rutgers University and Kessler Foundation and all methods were performed in accordance with the relevant guidelines and regulations. All participants provided written informed consent, and all were remunerated $100 for each testing session.

**Table 1:** The demographics of the sample. Age and Education are reported in years; MFIS_cognitive_ refers to the cognitive subscale of the Modified Fatigue Impact Scale; Depression refers to the score on the Chicago Multiscale Depression Inventory total score; Disease Duration (MS) and Time Since Injury (TBI) is reported in months.

	Control (N = 30)	MS (N = 31)	TBI (N = 31)	Overall (N = 92)
Age				
Mean [95% CI]	39.2 [35.0, 43.4]	47.9 [43.8, 52.1] *	42.9 [38.7, 47.0]	43.4 [40.9, 45.9]
Median [Min, Max]	38.0 [20.0, 58.0]	50.0 [26.0, 63.0]	46.0 [20.0, 65.0]	45.0 [20.0, 65.0]
Education				
Mean [95% CI]	15.6 [14.7, 16.4]	15.9 [15.1, 16.7]	14.8 [14.0, 15.6]	15.4 [14.9, 15.9]
Median [Min, Max]	16.0 [12.0, 20.0]	16.0 [12.0, 21.0]	14.0 [12.0, 20.0]	16.0 [12.0, 21.0]
Sex (biological)				
Women	17 (56.7%)	28 (90.3%) *	7 (22.6%) *†	52 (56.5%)
Men	13 (43.3%)	3 (9.7%) *	24 (77.4%) *†	40 (43.5%)
MFIS_cognitive_				
Mean [95% CI]	4.29 [0.6, 8.0]	20.4 [17.1, 23.6] *	17.7 [14.0, 21.4] *	14.7 [12.0, 17.3]
Median [Min, Max]	2.00 [0, 15.0]	20.0 [0, 35.0]	20.0 [0, 33.0]	14.0 [0, 35.0]
Depression				
Mean [95% CI]	43.6 [38.5, 48.8]	53.6 [49.1, 58.2] *	55.8 [51.0, 60.7] *	51.5 [48.5, 54.5]
Median [Min, Max]	41.5 [34.8, 64.4]	52.5 [40.7, 73.6]	51.5 [35.3, 87.6]	47.7 [34.8, 87.6]
Disease duration/Time since injury (mo)				
Mean [95% CI]		135 [108.5, 162.4]	90.8 [65.9, 115.8]	
Median [Min, Max]		132 [12.0, 336]	86.0 [14.0, 269]	
TBI severity				
Severe			28 (90.3%)	
Moderate/Severe			1 (3.2%)	
Complicated Mild			1 (3.2%)	
Mild			1 (3.2%)	
MS disease course				
Relapsing remitting		26 (83.9%)		
Progressive		5 (16.1%)		

*Symbol represents a statistically significant difference from the Control group.

^†^Symbol represents a statistically significant difference between the MS and TBI groups.

Individuals in the MS group were recruited from local MS clinics and the North Jersey Chapter of the National MS Society; all had a diagnosis of clinically definite MS^41^ (mean disease duration = 135 months, with a majority having a relapsing–remitting disease course) and were exacerbation-free for at least 4 weeks prior to testing. For the TBI group severity was defined as the lowest Glasgow Coma Scale (GCS) rating in the first 24 h following injury^34^. When a GCS score was not available, subjects were included only when there was sufficient medical documentation that allowed for a post-hoc estimation of initial GCS, or if other confirmatory data (e.g., positive anatomic neuroimaging findings, focal neurologic signs) were available. The mean time since injury for the TBI group was 90.8 months, and the majority had experienced a severe TBI. The majority of TBIs were due to motor vehicle accidents (71%), followed by falls (23%) and assault (6%).

### Behavioral data and paradigms

E-Prime software^35^ was used to present stimuli and record responses for both tasks. The order of the two tasks was counterbalanced across participants, and approximately one week elapsed between testing sessions. The outcome variables from the behavioral tasks were RT and accuracy. For the analyses of the RT and fMRI data, only correct trials were included.

### Working memory task

The 2-back condition of the n-back working memory paradigm was used to induce CF^36^, based on the literature showing working memory deficits following TBI^37,38^. Participants completed four blocks of the task, during which behavioral and fMRI data were acquired. To ensure all participants had comparable levels of proficiency, the task was practiced to criterion (80% correct) prior to scanning. Each block was comprised of 65 trials during which a single letter was presented at the center of the screen and participants were asked to press a button with their right index finger every time the letter was the same as the letter presented two trials previously in the sequence (e.g., R N Q N…). All participants were asked to respond as quickly as possible without sacrificing accuracy. Letters (A B C D F H J K M N P Q R S T V Z, presented with equal frequency) were presented in white on a black background (Arial 72-point font), and remained on the screen for 1.5 s, followed by an inter-trial interval (ITI) of 500 ms during which a white fixation cross was presented. The time between successive trials was jittered to optimize later deconvolution of the fMRI data using the Optseq2 program (https://surfer.nmr.mgh.harvard.edu/optseq/). The jittering was achieved by adding between zero and six 2-s-long intervals to each ITI. The intervals were drawn from a power distribution such that most were zero (i.e., ITI = 500 ms), followed by one (i.e., ITI = 2 s) and so on. The average ITI was 1587.9 ms (± 1769.7 SD), and each block lasted 4 min. 30 s.

### Processing speed task

The modified Symbol-Digit Modalities Test (mSDMT) was used as the other CF-induction task because of the centrality of processing speed deficits in MS^16,39^. All participants practiced the task to criterion (80% correct) to ensure proficiency before working through four blocks inside the scanner while fMRI data were acquired.

On each trial of the mSDMT, participants were presented with a reference grid at the top of the screen that had two rows and nine columns^23^. In the top row the numbers 1–9 were presented, and nine unique symbols were presented in the row beneath. In the bottom part of the screen a probe stimulus was presented that consisted of one number and one symbol. Subjects reported whether the probe number-symbol pair matched a number-symbol pair in the reference grid. Each trial lasted 4 s, and 55 trials were presented in each block. The Optseq2 was used to optimize the trial sequence by inserting between zero and six 2-s-long intervals between the mSDMT trials. The average ITI was 6139.7 ms (± 3544.2 SD), and each block lasted 7 min. 50 s.

### State CF

State CF was assessed at baseline and after each block of the two tasks using a visual analogue scale of fatigue (VAS-F)^40,41^. Participants were asked: “How mentally fatigued are you right now?” and rated their CF on a scale from 0 to 100 (with 0 being not at all fatigued and 100 being maximally fatigued) by reporting a numerical value to the experimenter.

### Neuroimaging acquisition

A 3-Tesla Siemens Allegra scanner was used to collect structural and functional MRI data. During each task block, a T2*-weighted Echo Planar sequence was used (echo time = 30 ms; repetition time = 2000 ms; field of view = 22 cm; flip angle = 80°; slice thickness = 4 mm, 32 slices, matrix = 64 × 64, in-plane resolution = 3.438 × 3.438 mm; number of acquisitions = 140 [2-back task] or 240 [mSDMT]). A high-resolution magnetization prepared rapid gradient echo (MPRAGE) image was also acquired (TE = 4.38 ms; TR = 2000 ms, FOV = 220 mm; flip angle = 8°; slice thickness = 1 mm, NEX = 1, matrix = 256 × 256, in-plane resolution = 0.859 × 0.859 mm^2^) and was used to register the functional data into standard MNI space for group analysis.

### Thalamic atrophy (TA) and caudate nucleus atrophy

TA was used as an index of cumulative damage to the central nervous system (CNS) based on both the MS^61^ and TBI^43,44^ literatures. Because of the central role the thalamus plays in brain function, TA represents a biomarker of neurodegeneration and associated dysfunction/decline^45^, and has been shown to be more sensitive to neurodegeneration early in the course of diseases such as MS than other, more global measures^46^. Because we had an hypothesis about the caudate nucleus, we also investigated atrophy of the caudate nucleus. As a manipulation check, we also calculated another measure of brain atrophy – brain parenchymal fraction (BPF) – for each participant to ensure that TA and BPF produced congruent results.

FreeSurfer^47^ was used to segment each participant’s MPRAGE, resulting in an estimate of right and left thalamic and caudate volumes as well as BPF. Thalamic and caudate volumes were converted into z-scores using publicly available age-, sex-, and intracranial volume-adjusted normative values for thalamic volume^48^. The z-scores were then averaged to estimate each participant’s TA and caudate atrophy (CA), where positive z-scores represented larger than expected volume (in relation to the normative sample) and negative values represented smaller than expected volumes.

### Analyses

Statistical analyses were conducted using the R statistical package (version 3.4.3). Prior to analysis, the normality of all variables was assessed by visual inspection and the Agostino test^49^. In those cases when the data were found to be skewed, they were transformed using the Box Cox method^50^.

#### Demographics

Differences between the groups in age, education and depression were tested with one-way ANOVAs. The factor was Group (MS, TBI, control). Differences in sex across the groups was assessed using a Chi-square test. All statistically significant differences were included in the group level analyses as covariates.

#### State CF

For the analysis of the VAS-F scores, a Linear Mixed Effects analysis (LME) was used. Group (MS, TBI, control) and Sex (men vs. women) were between-subjects factors; Task (2-back vs. mSDMT) and Rating (VAS-F rating acquired at baseline and after each task: 5 ratings per task) were fixed effects; age was a quantitative variable; and Participant was included as a random factor.

For analyses in which we were interested in the relationship between CF and another dependent measure (RT, accuracy and functional activation), the VAS-F scores obtained before and after each task block were averaged to provide an estimate of the amount of CF during each block. Because the VAS-F scores were skewed, they were transformed before being mean-centered for each group.

#### Rate of CF increase and stability of CF across task

To investigate the stability of the rate of increase in CF across the four runs of the two tasks, a regression line was fit to the five VAS-F scores for each task and participant^51^. The slope of this regression line was operationalized as the CF rate (CFR) and the correlation of the CFR across tasks was calculated with a Pearson’s correlation. Additionally, the extent to which the CFR differed across participants was investigated using an LME with the factors of Group, and Task (as above), with Sex and Age included as covariates.

Similarly, the stability of participants’ CF level prior to undertaking the task was assessed by correlating the intercept of the regression line calculated for each task and participant. And, as with the slope data, an LME model was used to assess whether the intercept varied across groups and tasks.

#### The relationship between CF and behavioral performance

Response time and accuracy were analyzed with an LME that included the factors of Group (MS, TBI, control), Task (2-back, mSDMT), Run (runs 1–4), and Sex (women vs. men); the VAS-F scores and age were included as quantitative variables; participant was included as a random factor. For RT, only RTs from correct trials were included in the analysis. Accuracy was calculated as the number of trials on which the correct response in each block was made divided by the total number of trials in that block.

#### The relationship between state CF and trait CF

To investigate whether state and trait measures of CF were related to one another, two linear regressions were used. VAS-F scores were used as the measure of state CF and the cognitive subscale of the Modified Fatigue Impact Scale (MFIS_cognitive_) was used as the measure for trait CF. One regression was run for each task (2-back and mSDMT), and in each the average VAS-F score (across the five ratings) was predicted by MFIS_cognitive_ score. Group, age and sex were also included in each model. As a manipulation check, a one-way ANOVA was also used to test for differences in MFIS_cognitive_ across the groups; age and sex were included as covariates.

#### The relationship between CF and brain atrophy

One-way ANOVAs were used to test whether TA and CA differed across groups. ANCOVAs were then used to test whether there was a relationship between state CF and TA and between state CF and CA. The factors for this ANCOVA were Group (MS vs. TBI), and Sex (men vs. women); and the VAS-F scores (averaged across the runs) and age were included as quantitative variables. The same ANCOVA was used for the BPF data.

#### The relationship between CF and brain activation

fMRI: The neuroimaging data were preprocessed using *fMRIPrep* 1.4.1 ^RRID:SCR_016216,^^52^, which is based on *Nipype* 1.2.0 ^RRID:SCR_002502,^^53^. This included segmentation and warping of the T1-weighted anatomical scan into standard space (using the MNI template). The functional MRI data was motion-corrected, smoothed, scaled and warped into the same standard anatomical space. The details of these preprocessing steps can be found in the supplemental data section.

An LME was used for a whole-brain analysis (3dLMEr from the AFNI suite of processing tools) with Task (2-back vs. mSDMT), Sex (men vs. women) and Run (runs 1–4) as factors; the VAS-F scores, and age were included as quantitative variables; participant was included as a random factor. As in previous work^15,21,54,55^ only runs resulting in CF were included in the analysis (i.e., runs in which the VAS-F score was greater than zero). This resulted in the exclusion of ~ 20% of runs (see supplemental data for details).

The results of this analysis were corrected for multiple comparisons by using an individual voxel probability threshold of *p* < 0.005 and a cluster threshold of 28 voxels (voxel dimension = 2.4 × 2.4 × 4 mm). Monte Carlo simulations, using 3dClustSim (version AFNI_21.3.04, compile date: Oct 20, 2021) showed this combination resulted in a corrected whole brain alpha level of *p* < 0.05. Using the same approach, we calculated the corrected alpha level for the striatum and thalamus (about which we had prior hypotheses) to be nine voxels.

#### In-scanner movement

Differences in in-scanner movement were assessed by identifying the maximum framewise displacement (FD) from the realignment step for each run of each task for each participant, exclusive of those frames that were censored from the deconvolution (first level analysis). The remaining FD data were analyzed with an LME that included the factors of Group (MS, TBI, control), Task (2-back, mSDMT), Run (runs 1–4), and Sex (women vs. men); age was a quantitative variable and participant was included as a random factor.

## Results

### Demographics

As Table 1 shows, the groups significantly differed in age (F(2,89) = 4.32, *p* = 0.02, η^2^ = 0.09), with the only pairwise difference being between the MS and control groups (*p* = 0.01, 95% CI [1.6, 15.9]). There were no differences in education between the groups. The distribution of men and women differed in the three groups (Χ^2^(2) = 28.94, *p* < 0.001, effect size [Cramer’s V] = 0.56). The MS group had more women while the TBI group had more men, as expected^25,26^. As in previous literature^30,56^, there was a significant difference in depression across the groups (F(2,77) = 7.63, *p* = 0.002, η^2^ = 0.17), with both the MS and TBI groups reporting significantly higher depression than the Control group (MS-Control: difference = 10.0, *p* = 0.01, 95% CI [1.72, 18.29]; TBI-Control: difference = 12.17, p = 0.003, 95% CI [3.66, 20.68]), but not differing from one another. In all subsequent analyses age, sex and depression were included as covariates.

### State CF

**Figure 1:**
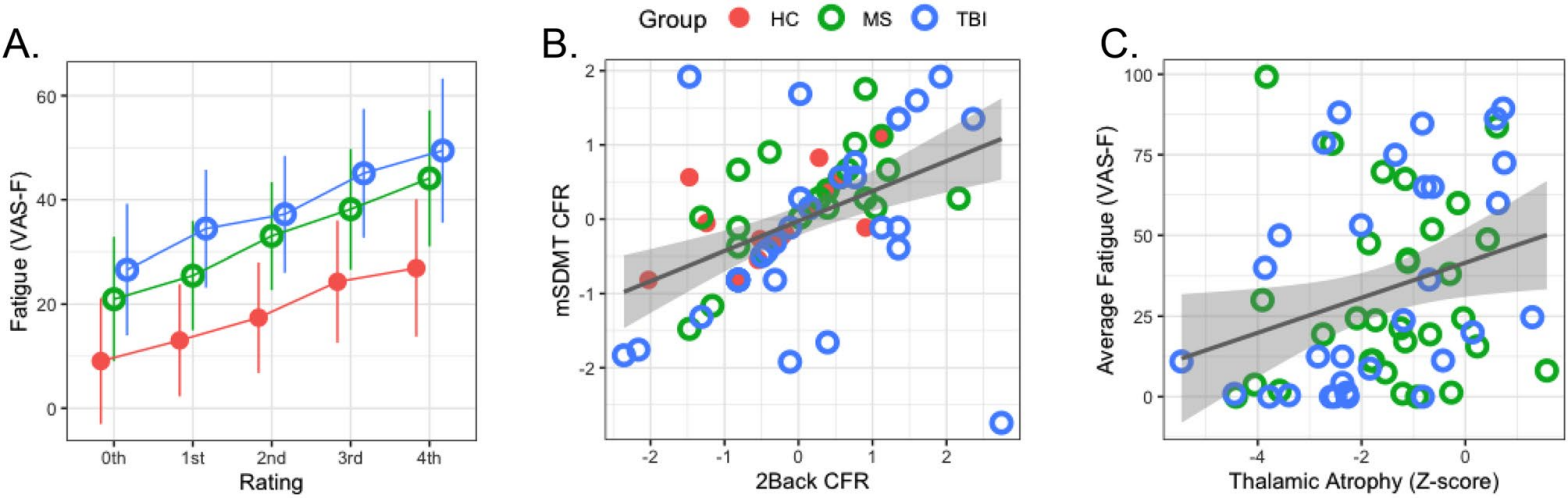
The effects of cognitively fatiguing tasks on ratings of fatigue (VAS-F scores). (**A**) The VAS-F scores for the three groups (HC shown in red filled circles, MS shown in green open circles and TBI shown in blue open circles) for each of the five ratings. The 0th rating was collected prior to the first run of the fatigue induction task (the 2-back or the mSDMT task); the 1st rating was collected after the first run of the fatigue induction task; the 2nd rating was collected after the second run, and so on. Error bars represent standard error of the mean. (**B**) The significant positive correlation between the Cognitive Fatigue Rate (CFR) on the 2-back task and the mSDMT. (**C**) The significant positive correlation between brain atrophy, operationalized as thalamic atrophy, and the average VAS-F rating for the MS (green open circles) and the TBI (blue open circles) groups. For panels B and C the shaded areas represent 95% confidence intervals.

Visual inspection of Fig. 1A suggests that there were differences in VAS-F scores between the groups, that the VAS-F scores increased across successive ratings, and that these two factors (Group and Rating) did not interact. The results supported this interpretation. The main effect of Group was significant (F(2,77) = 3.63, *p* = 0.03, $\eta_{{{\text{partial}}}}^{{2}}$ = 0.09): controls reported the least CF, followed by individuals with MS, and individuals who had sustained a TBI reported the most (see Fig. 1A). Of the pairwise differences, the Control group (95% CI [7.6, 28.9]) differed significantly from the TBI group (95% CI [28.0, 49.3]) (t(77.9) = 2.49, *p* = 0.01) and trended toward significance from the MS group (95% CI [21.7, 43.2]) (t(76.7) = 1.89, *p* = 0.06); the MS and TBI groups did not significantly differ from one another. The main effect of Task was significant (F(1,630.4) = 4.00, *p* = 0.05, $\eta_{{{\text{partial}}}}^{{2}}$ = 0.006), which was due to higher VAS-F ratings for the mSDMT task (31.5) than for the 2-back task (28.1). The main effect of Rating was significant (F(4,222.9) = 2.50, *p* = 0.045, $\eta_{{{\text{partial}}}}^{{2}}$ = 0.04) which was due to all three groups reporting increasing CF over time (i.e., across the five VAS-F ratings: 19.0, 24.4, 29.3, 36.0, 40.3; see Fig. 1A). No other effects or interactions reached conventional levels of significance.

### Rate of CF increase and stability of CF across task

There was a significant positive correlation between CFR across the two tasks (r = 0.50, *p* < 0.001) (see Fig. 1B): high CFR on the 2-back task was associated with a high CFR on the mSDMT. There was also a significant positive correlation between the intercepts across the two tasks (r = 0.70, *p* < 0.001) (see Figure S1): a high intercept for the 2-back task was associated with a high intercept for the mSDMT. The LME testing whether the three groups differed on CFR showed no significant effects. In contrast, the result of the LME of the intercept data showed a significant effect of Group (F(2,74) = 3.18, *p* = 0.047, $\eta_{{{\text{partial}}}}^{{2}}$ = 0.08). The intercept, or baseline level of CF, of the control group was lowest (mean = 14.3, 95% CI [4.3, 24.4]), the intercept of the MS group was higher (mean = 26.4, 95% CI [16.2, 36.5]) and the intercept of the TBI group was highest (mean = 32.8, 95% CI [22.8, 42.8]). This result was consistent with the VAS-F data shown in Fig. 1A. Of the pairwise comparisons, only the difference between the TBI and control groups was significant (t(74) = 2.43, *p* = 0.046).

### The relationship between CF and behavioral performance

For RT, there was a main effect of Group (F(2,58.1) = 3.32, *p* = 0.04, $\eta_{{{\text{partial}}}}^{{2}}$ = 0.10): controls responded with the shortest latencies (1191 ms, 95% CI [1092, 1289]), while individuals with MS (1346 ms, 95% CI [1256, 1437]) and individuals who had sustained a TBI responded with longer latencies (1342 ms, 95% CI [1249, 1434)). There was a main effect of Task (F(1,371.0) > 100, *p* < 0.001, $\eta_{{{\text{partial}}}}^{{2}}$ = 0.87): participants responded with longer latencies on the mSDMT (1759 ms, 95% CI [1706, 1812]) than on the 2-back task (827 ms, 95% CI [775, 879]). The relationship between VAS-F and RT was also significant (F(1, 190.6) = 5.03, *p* < 0.05, $\eta_{{{\text{partial}}}}^{{2}}$ = 0.03) and was positive: as VAS-F increased, participants responded with longer latencies (coefficient = 1.56). Finally, there was an interaction between Group and Task (F(2, 371.72) = 22.97, *p* < 0.001, $\eta_{{{\text{partial}}}}^{{2}}$ = 0.11). As shown in Figure S2, the control group responded with shorter latencies than the other two groups on the mSDMT (*p* < 0.05; the latencies for the MS and TBI groups were not significantly different), while on the 2-back task the three groups responded with comparable latencies (*p* > 0.05). No other effects or interactions were significant.

All three groups performed the tasks with greater than 90% accuracy. There was nevertheless a main effect of Group (F(2, 64.7) = 4.69, *p* = 0.01, $\eta_{{{\text{partial}}}}^{{2}}$ = 0.13): controls responded with the most accuracy (95.6% [0.956], 95% CI [0.94, 0.98]), individuals with MS (91.9% [0.919], 95% CI [0.90, 0.94]) and TBI (92.5% [0.925], 95% CI [0.91, 0.94]) responded with somewhat lower accuracy. Only the difference between the MS and Control group was significant (p < 0.05). The main effect of Task was significant (F(1, 381.8) > 100, *p* < 0.001, $\eta_{{{\text{partial}}}}^{{2}}$ = 0.45) and was due to participants responding with more accuracy on the mSDMT (97.0% [0.97], 95% CI [0.96, 0.98]) than the 2-back task (89.7% [0.897], 95% CI [0.89, 0.91)). There was an interaction between Group and Task (F(2, 382.3) = 13.74, *p* < 0.001, $\eta_{{{\text{partial}}}}^{{2}}$ = 0.07). This can be seen in Figure S2 and was due to all three groups being comparably accurate on the mSDMT task (*p* > 0.05 for all pairwise comparisons), but for the 2-back task the control group was more accurate than the MS or the TBI groups (*p* < 0.01 in both cases; the accuracy of MS and TBI groups did not significantly differ). There was also an interaction between Task and VAS-F (F(1, 390.6) = 6.21, *p* = 0.01, $\eta_{{{\text{partial}}}}^{{2}}$ = 0.02). For both tasks there was a negative relationship between VAS-F and accuracy and the relationship was larger for the 2-back task (coefficient =  − 0.03) than for the mSDMT (− 0.0000009). Finally, the three-way interaction between Group, Task and VAS-F was significant (F(2, 387.9) = 3.70, *p* = 0.025, $\eta_{{{\text{partial}}}}^{{2}}$ = 0.02). As shown in Figure S3, this was due to the relationship between accuracy and CF being negative for both tasks for the Control group, while for the MS group the relationship was negative for the 2-back task but positive for the mSDMT and for the TBI group, the relationship was slightly positive for both tasks (the coefficients for each Group-Task pairing are shown in Figure S3). Of the pairwise comparisons, the only significant difference was between the slopes for the two tasks in the MS group (*p* < 0.01).

### The relationship between state CF and trait CF

The relationship between VAS-F and MFIS_cognitive_ was not significant for either task. However, as Table 1 suggests, there were significant differences in MFIS_cognitive_ across the groups (F(2,65) = 20.50, *p* < 0.001, $\eta_{{{\text{partial}}}}^{{2}}$ = 0.39), with both the MS and TBI group reporting significantly more trait CF than the Control group (in both cases, t(65) ≥ 5.37, *p* < 0.001). The MS and TBI groups did not significantly differ in their MFIS_cognitive_ scores (t(65) = 0.17).

### The relationship between CF and brain atrophy

In the TA data, there was a significant effect of Group (F(2,88) = 24.60, *p* < 0.001, η^2^ = 0.36): both the MS (mean Z =  − 1.44, 95% CI [-1.93, − 0.95]) and TBI (mean Z =  − 1.72, 95% CI [− 2.21, − 1.23]) groups had more TA than controls (mean Z = 0.58, 95% CI [0.074, 1.09]) (t(88) ≥ 5.68, *p* < 0.001). There was no significant difference in TA between the MS and TBI groups. Similarly, in the CA data, there was a significant effect of Group (F(2,88) = 9.12, *p* < 0.001, η^2^ = 0.17) because both the MS (mean Z =  − 0.23, 95% CI [− 0.63, 0.17]) and TBI (mean Z =  − 0.32, 95% CI [− 0.72, 0.08]) groups had more CA than controls (mean Z = 0.80, 95% CI [0.39, 1.21]) (t(88) ≥ 3.55, *p* < 0.007). The same analysis applied to the BPF data yielded the same pattern of results. Indeed, the correlation between BPF and normalized thalamic volume was r = 0.93 (t(90) = 24.67, *p* < 0.001) and between BPF and normalized caudate volume was r = 0.87 (t(90) = 16.643, *p* < 0.001).

While the MS and TBI groups had similar levels of TA and CA, the relationship between these measures of brain atrophy and state CF may nevertheless have differed between the two groups. To test this, we analyzed the relationship between state CF and TA, accounting for Group (MS vs. TBI), age and sex. This analysis showed a significant effect of TA on VAS-F (F(1,56) = 5.84, *p* = 0.02, $\eta_{{{\text{partial}}}}^{{2}}$ = 0.09) which was due to a positive relationship (coefficient = 5.93): the more TA there was, the less CF was reported (see Fig. 1C). No other effects or interactions were significant, suggesting that the relationship between CF and TA was similar for the MS and TBI groups. The same analysis was run on the CA data and yielded no significant results.

### The relationship between CF and brain activation

#### In-scanner movement

The main effect of Group was not significant in the analysis of the FD data, nor did it interact with any of the other variables.

#### fMRI

**Figure 2:**
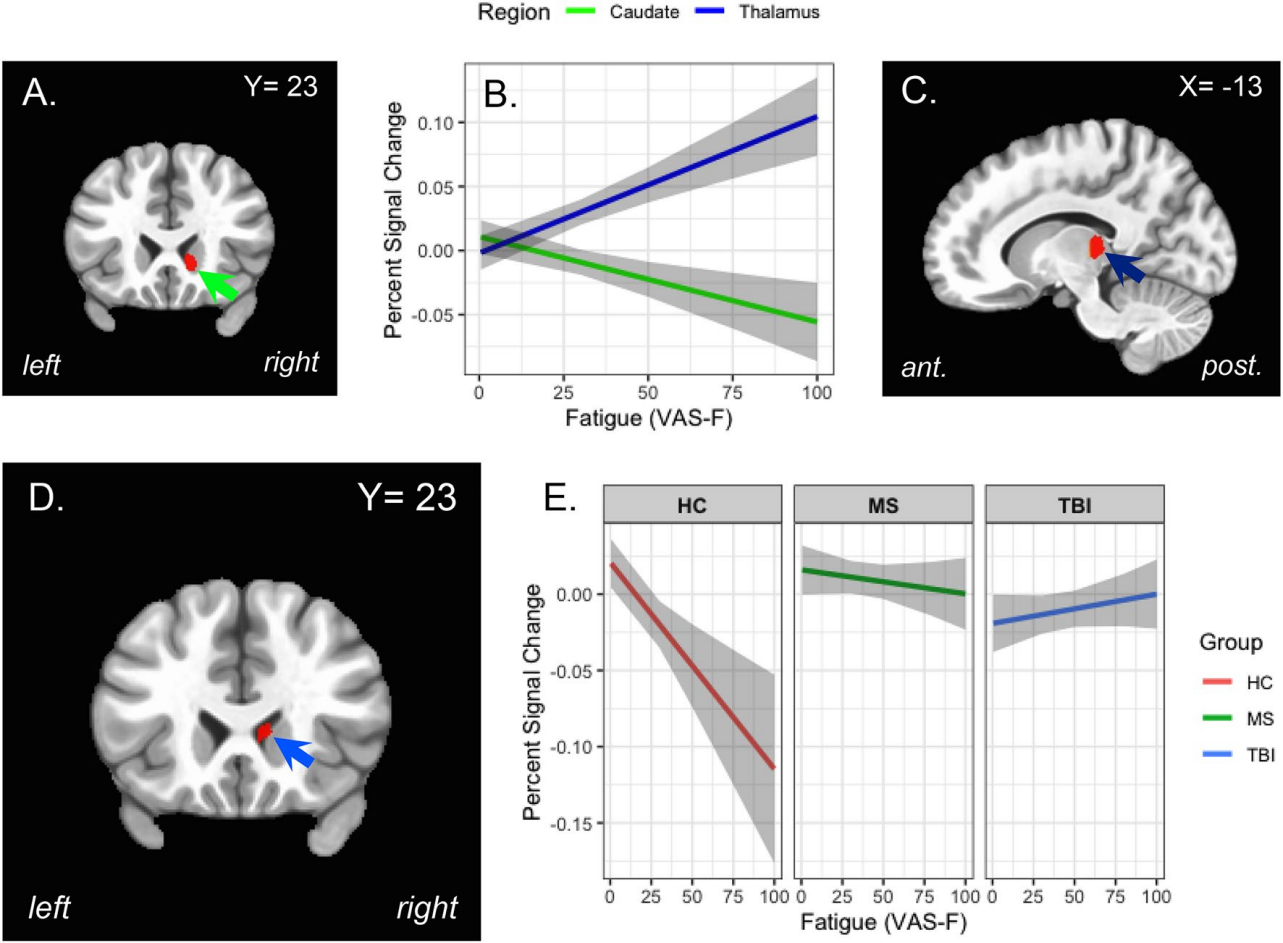
The effects of cognitively fatiguing tasks on brain activation. (**A**) The location in the caudate of brain activation showing the main effect of CF (green arrow). (**B**) The significant negative relationship between the CF (VAS-F scores) and brain activation in the caudate nucleus (green line) and the significant positive relationship between CF and brain activation in the thalamus (dark blue line). (**C**) The location of thalamic brain activation showing the main effect of CF (dark blue arrow). (**D**) The location of brain activation showing at interaction between Group (HC, MS and TBI) and CF: the caudate nucleus of the basal ganglia (blue arrow). (**E**) Activation in the caudate nucleus as a function of CF for each of the three groups; Controls are shown in red, the MS group in green and the TBI group in blue. For panels B and E the shaded areas represent 95% confidence intervals.

There was a main effect of state CF (VAS-F) in the thalamus and the caudate nucleus (see Table 2 and Fig. 2A,C). As Fig. 2B shows, the relationship was complimentary, with activation in the thalamus increasing as CF increased and activation in the caudate decreasing as CF increased (see also Table 2).

**Table 2:** The brain areas associated with the main effect of CF. X Y Z = the location of the voxel with peak intensity in each cluster; Vox refers to the number of voxels in the cluster; **Χ**^2^ Stat refers to the maximal **Χ**^2^ statistic in each cluster. Coefficient refers to the slope of the best fitting linear relationship between brain activation and VAS-F.

Location	X	Y	Z	Vox	Χ^2^ Stat	Coefficient
Caudate nucleus	7	16	− 6	25	20.59	− 0.0007
Thalamus	− 13	− 29	6.0	18	17.28	0.0011

The effect of VAS-F was modified by Group in the caudate nucleus of the basal ganglia (see Table 3 and Fig. 2D,E). As Fig. 2E shows, there was a negative relationship between activation in the caudate and VAS-F for the Control group, a weakly negative relationship for the MS group and a positive relationship for the TBI group. This pattern was also evident in another area more dorsal in the caudate nucleus (see Table 3).

**Table 3:** The brain areas associated with the interaction of Group and VAS-F. X Y Z = the location of the voxel with peak intensity in each cluster; Vox refers to the number of voxels in the cluster; **Χ**^2^ Stat refers to the maximal **Χ**^2^ statistic in each cluster. The slope of the best fitting linear relationship (the coefficient) between brain activation and VAS-F for each group are shown at the right.

Location	X	Y	Z	Vox	Χ^2^ Stat	Coefficients		
						Control	MS	TBI
Caudate nucleus	7	19	10	10	17.98	− 0.0012	− 0.0002	0.0002
Caudate nucleus	14	9	22	9	16.28	− 0.0017	− 0.0003	0.0001

For completeness, task related activation and differences are shown in Figure S4 and Table S2.

## Discussion

This is the first study to compare CF and its neural underpinnings across clinical groups and across tasks. We expected the MS and TBI groups to report comparable levels of CF that were significantly greater than the CF reported by the control group. The results largely supported these hypotheses, with the MS and TBI groups reporting higher levels of fatigue than the control group (the difference between the TBI and control group was significant at *p* < 0.05 while the difference between the MS and control group only trended towards significance at *p* = 0.06). These differences provide support to the idea that CF experienced by clinical groups is similar and that this ‘pathological’ CF differs from the CF experienced by the control group.

While the clinical groups reported more CF than controls, the rate of increase of CF was comparable across the three groups as we had hypothesized. That is, brain injury or disease resulted in a shift of the baseline level of CF (as shown by the differences in the intercept of the regression line fitted to the VAS-F scores; see also Fig. 1A), but did not affect the rate at which state CF accrues during task performance. This suggests that higher CF reported by clinical samples (‘pathological fatigue’) results from a baseline shift rather than from different accrual rates of CF during task performance.

In previous work, we have argued that CF is related to a change in the balance between effort and reward^54,57,58^ That is, CF is related to maintaining an aspect of homeostasis whereby the effort expended merits the reward received. This view is supported by the behavioral data in the current study: for all three groups, higher VAS-F scores were associated with longer latencies as hypothesized, suggesting gradual exhaustion of cognitive resources with repeated task performance. It is also supported by the positive relationship between greater TA and lower VAS-F scores in the clinical groups, as well as by the fMRI data, wherein higher VAS-F scores were associated with less activation in the caudate nucleus and more in the thalamus. These results accord well with what is known of striato-thalamic connections. Not only does the striatum project to the thalamus^59^, but the connections are inhibitory such that more activation in the striatum would be expected to result in less activation in the thalamus^59^.

Our results show some of the important nuances of CF in persons who have sustained a brain injury or have a neurological disease. Individuals with TBI and MS did not differ in TA, suggesting a certain amount of similarity in the extent to which CNS damage resulted in widespread neuronal degradation. Moreover, individuals with TBI and MS did not differ in CFR, nor did each group’s CFR differ from the control group, suggesting generality in the accrual of CF over time. Finally, individuals with TBI and MS did not differ in the amount of CF they reported, and individuals with TBI reported significantly more CF than Controls. The fMRI results accord well with the similarities and differences in the behavioral results. For example, the CF-related activation in the thalamus and the ventral aspect of the caudate nucleus was common to all three groups and both tasks, which may reflect common aspects of CF such as CFR. Furthermore, there were differences across the groups in the activation more dorsally in the caudate nucleus, which may help explain the differences in the intensity of CF across the groups. For the Control group, more activation in this region was associated with less CF, which was similar to the relationship between activation and CF for all three groups more ventrally in the caudate. However, for the TBI group, who reported the most CF, this relationship was flipped such that more activation was associated with more CF. This result is compatible with idea that TBI results in disruption of the dopaminergic system, which contributes to CF^58^. The findings from the MS group are also compatible with this hypothesis inasmuch as the relationship between activation in the caudate nucleus and CF falls between that of the Control and TBI groups and they report a numerically intermediate level of CF (the difference between the Control and MS groups in CF trended toward significance). Furthermore, the finding that the group-level differences in activation in the caudate nucleus was found more dorsally in the caudate (relative to the more ventral caudate region that showed CF-related activation in all three groups) is consistent with literature showing a ventral-dorsal gradient in dopamine expression (with more expressed more dorsally) in the caudate nucleus^60^. Finally, the finding that different regions within the caudate nucleus showed differential CF-related activation may help to explain why caudate nucleus atrophy was not significantly related to VAS-F scores where thalamic atrophy was. Because different regions of the caudate nucleus responded differently to CF, the volume of the caudate nucleus may be more variable than the thalamus which did not show such CF-related regional differences.

### Stability of VAS-F ratings

The results of the analyses of the VAS-F data also suggest that there was a great deal of stability in CF ratings across the three groups despite substantial differences between the tasks used to induce CF in terms of stimulation, cognitive processes/demands and task length. The rate of CF increase (CFR) was strongly correlated across the two tasks, as was the intercept of the regression line fitted to the VAS-F scores. This suggests that the VAS-F is a robust and replicable measure of state CF during neurocognitive tasks that should be tested using other fMRI paradigms. Moreover, the fact that the VAS-F did not correlate with our measure of trait CF suggests that state and trait measures may assess different constructs, as has been shown previously^9,61^.

### Limitations

The conclusions we can draw from this study are limited in several ways. Our sample of ~ 30 individuals in each group is relatively small, and the results reported here should be replicated in a different, larger sample. Furthermore, our sample was not matched on variables such as age and biological sex because this was a secondary analysis of two datasets. Given the demographic differences associated with MS and TBI, differences in sex were expected, but it would be reassuring to replicate our results in a sample that was matched across all relevant variables. While age was included in all analyses as a covariate, age has been correlated with decreases in CF^62^, suggesting that our somewhat older MS group might have reported less CF than a younger cohort. Similarly, it is not clear that the MS and TBI groups were matched on severity of disease/injury. The groups did not differ in terms of TA, but it may nevertheless have been the case that the damage to the CNS in the MS group was less than in the TBI group. Finally, it would have been ideal to have had diffusion weighted data to assess axonal damage in our MS and TBI groups inasmuch as both MS and TBI affect white matter.

### Conclusions

This is the first study to systematically investigate the effects of inducing CF using two different tasks in three different populations of participants. Our results show that individuals who have sustained a TBI report approximately the same amount of CF as individuals with MS, and more CF than controls (while the difference between the MS and control groups trended towards significance). The CF experienced during the two tasks showed significant stability, both in terms of the slope of the increase in CF across time and in the baseline level of CF reported. Cognitive fatigue was related to TA such that more atrophy was associated with less CF, lending support to the idea that the purpose of CF is to maintain homeostasis in the cognitive system. Brain activation in the thalamus and the caudate nucleus were related to CF across the three groups while CF-related activation more dorsally in the caudate differed across the groups. These results suggest that the thalamus and caudate may play a central role in CF and that more dorsally the role played by the caudate may be sensitive to damage in other areas of the cortex associated with particular types of insult.

### Supplementary Information

Supplementary Information.

## Data Availability

The data used in this study are available on request. Open data sharing is not possible, given the sensitive nature of participants’ data and the language in the informed consent that each participant signed. However, deidentified derivative data will be made available by the lead author (GRW) upon formal request indicating name and affiliation of the researcher as well as a brief description of the intended use for the data. All requests will be required to comply with Kessler Foundation procedures.
