# Supplementary material for: Evaluating the effects of brain injury, disease and tasks on cognitive fatigue
Source: Sci Rep. 2023 Nov 17;13:20166. doi: 10.1038/s41598-023-46918-y (PMC10656417; doi:10.1038/s41598-023-46918-y)
Supplement: Supplementary file 1 — Supplementary Information. [file 41598_2023_46918_MOESM1_ESM.docx]

**Neuroimaging preprocessing steps**

For anatomical preprocessing, the T1-weighted (T1w) image from each subject was corrected for intensity non-uniformity (INU) with N4BiasFieldCorrection [12], distributed with ANTs 2.2.0 [1, RRID:SCR_004757], and used as T1w-reference throughout the workflow. The T1w-reference was then skull-stripped with a *Nipype* implementation of the antsBrainExtraction.sh workflow (from ANTs), using OASIS30ANTs as target template. Brain tissue segmentation of cerebrospinal fluid (CSF), white-matter (WM) and gray-matter (GM) was performed on the brain-extracted T1w using fast (FSL 5.0.9, RRID:SCR_002823, [13]). Volume-based spatial normalization to one standard space (MNI152NLin2009cAsym) was performed through nonlinear registration with antsRegistration (ANTs 2.2.0), using brain-extracted versions of both T1w reference and the T1w template. The following template was selected for spatial normalization: *ICBM 152 Nonlinear Asymmetrical template version 2009c* [[4], RRID:SCR_008796; TemplateFlow ID: MNI152NLin2009cAsym].

The following preprocessing was performed for each of the eight BOLD runs for each participant (across the two tasks). First, a reference volume and its skull-stripped version were generated using a custom methodology of *fMRIPrep*. The BOLD reference was then co-registered to the T1w reference using flirt (FSL 5.0.9, [7]) with the boundary-based registration [5] cost-function. Co-registration was configured with nine degrees of freedom to account for distortions remaining in the BOLD reference. Head-motion parameters with respect to the BOLD reference (transformation matrices, and six corresponding rotation and translation parameters) are estimated before any spatiotemporal filtering using mcflirt (FSL 5.0.9, [6]). BOLD runs were slice-time corrected using 3dTshift from AFNI 20160207 ([3], RRID:SCR_005927). The BOLD time-series (including slice-timing correction when applied) were resampled onto their original, native space by applying a single, composite transform to correct for head-motion and susceptibility distortions. These resampled BOLD time-series will be referred to as *preprocessed BOLD in original space*, or just *preprocessed BOLD*. The BOLD time-series were resampled into standard space, generating a *preprocessed BOLD run in [‘MNI152NLin2009cAsym’] space*. First, a reference volume and its skull-stripped version were generated using a custom methodology of *fMRIPrep*. Several confounding time-series were calculated based on the *preprocessed BOLD*: framewise displacement (FD), DVARS and three region-wise global signals. FD and DVARS are calculated for each functional run, both using their implementations in *Nipype* (following the definitions by Power et al. [10]). The three global signals are extracted within the CSF, WM, and whole-brain masks. Additionally, a set of physiological regressors were extracted to allow for component-based noise correction (*CompCor*, [2]). Principal components are estimated after high-pass filtering the *preprocessed BOLD* time-series (using a discrete cosine filter with 128s cut-off) for the two *CompCor* variants: temporal (tCompCor) and anatomical (aCompCor). tCompCor components are then calculated from the top 5% variable voxels within a mask covering the subcortical regions. This subcortical mask is obtained by heavily eroding the brain mask, which ensures it does not include cortical GM regions. For aCompCor, components are calculated within the intersection of the aforementioned mask and the union of CSF and WM masks calculated in T1w space, after their projection to the native space of each functional run (using the inverse BOLD-to-T1w transformation). Components are also calculated separately within the WM and CSF masks. For each CompCor decomposition, the *k* components with the largest singular values are retained, such that the retained components’ time series are sufficient to explain 50 percent of variance across the nuisance mask (CSF, WM, combined, or temporal). The remaining components are dropped from consideration. The head-motion estimates calculated in the correction step were also placed within the corresponding confounds file. The confound time series derived from head motion estimates and global signals were expanded with the inclusion of temporal derivatives and quadratic terms for each [11]. Frames that exceeded a Euclidian norm of 0.4 mm were annotated as motion outliers. All resamplings can be performed with *a single interpolation step* by composing all the pertinent transformations (i.e. head-motion transform matrices, susceptibility distortion correction when available, and co-registrations to anatomical and output spaces). Gridded (volumetric) resamplings were performed using antsApplyTransforms (ANTs), configured with Lanczos interpolation to minimize the smoothing effects of other kernels [8]. Non-gridded (surface) resamplings were performed using mri_vol2surf (FreeSurfer).

The resulting data were then smoothed with an isometric 6mm Gaussian kernel, scaled to the grand mean intensity and deconvolved. Each block was deconvolved separately. In the deconvolution the following were included as regressors of no interest: a set of basis functions to model signal drift, the motion parameters and their derivatives, Framewise Displacement[9], the first six components from aCompCor (above). In addition, frames (TRs) exceeding a Euclidean norm of 0.4 mm and the immediately preceding frame were excluded from analysis. The regressors of interest were the correct trials of each block, and the coefficient of fit of the correct trials were entered into the group-level analysis.

**Number of blocks with and without CF**

|  | **Control (N=226)** | **MS (N=239)** | **TBI (N=247)** | **Overall (N=712)** |
| --- | --- | --- | --- | --- |
| **Fatigue presence** |  |  |  |  |
| CF reported | 173 (76.5%) | 212 (88.7%) | 185 (74.9%) | 570 (80.1%) |
| No CF reported | 53 (23.5%) | 27 (11.3%) | 62 (25.1%) | 142 (19.9%) |
| **Table S1. Blocks with and without CF.** The total number of blocks included for each group are shown at the top and the number and percentage of blocks with and without CF are reported below. | | | | |

A Χ^2^ test showed that the numbers of blocks with fatigue was different across the groups (Χ^2^(2)=17.05, p<0.001) with the Control group and the TBI group differing from the MS group ((Χ^2^(1)=11.21, p<0.001 and (Χ^2^(1)=14.56, p<0.0001, respectively), but not from one another (Χ^2^(1)=0.10, p>0.05). Regarding the number of participants in each group who reported experiencing zero CF on at least one run, there were 10 Controls, six individuals with MS and 11 individuals who had sustained a TBI.

**Results**

**Rate of CF increase and stability of CF across time**

Intercept of best-fitting regression line: Figure S1 shows the correlation of the intercepts of the best-fitting regression lines from the two tasks (2-back and mSDMT).

Figure S1. The positive correlation between the VAS-F intercept from the mSDMT and the 2-back tasks.


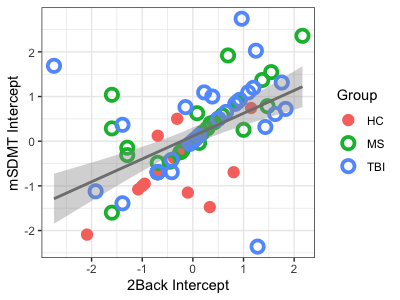


**The relationship between CF and behavioral performance**

RT and Accuracy: Figure S2 shows the RT and accuracy data for the two tasks (2-back, mSDMT), separately for the three groups (MS, TBI, Control).


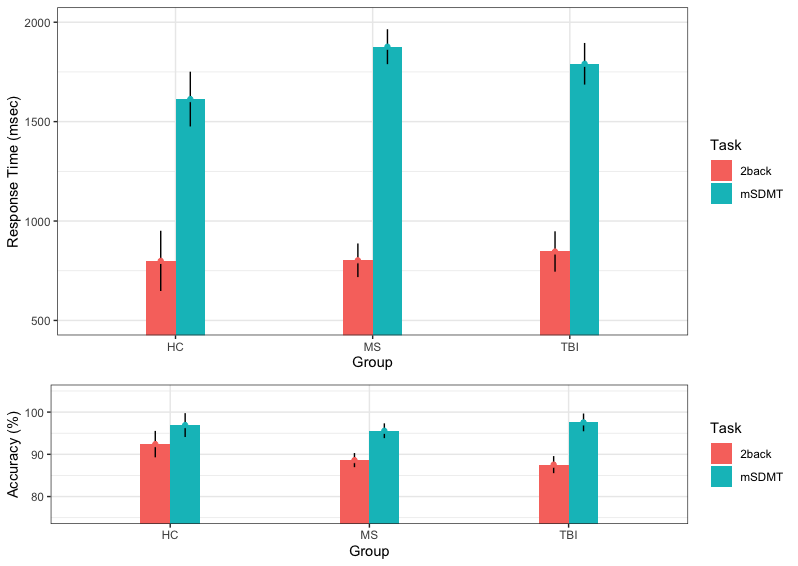


Figure S2. The interaction between Group and Task for RT (top) and accuracy (bottom).

|  | Slope | |  |
| --- | --- | --- | --- |
| Group | 2-back | mSDMT | p-value |
| HC | -0.05947 | -0.06523 | ns |
| MS | -0.05000 | 0.04473 | p<0.01 |
| TBI | 0.00624 | 0.02050 | ns |


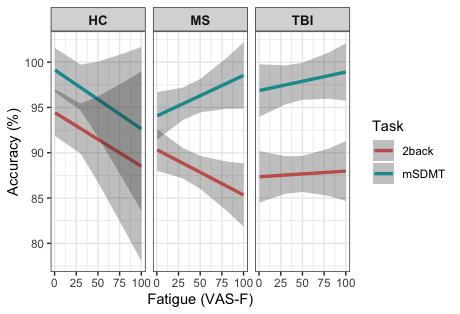


B.

A.

**Figure S3. The three-way interaction between Group, Task and VAS-F.** (A) The three groups are plotted in the three panels. The relationship between VAS-F and accuracy is plotted in red for the 2back task and in green for the mSDMT. The shaded areas represent 95% confidence intervals. (B) A table of the coefficients for each pairing of Group and Task.

**Task-related activation**

0.35

-0.35


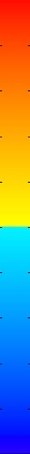


A. 2-back task

B. mSDMT task

C. 2-back – mSDMT


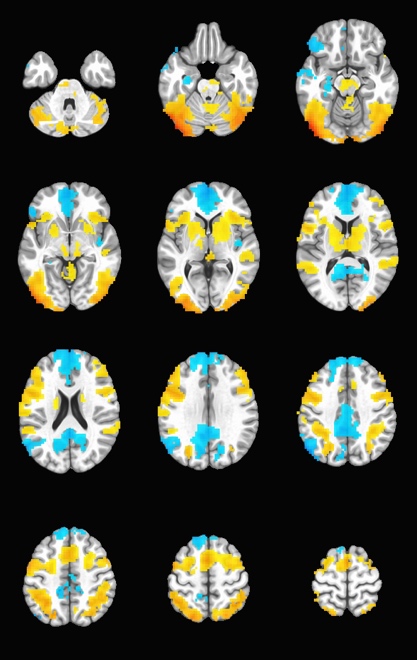

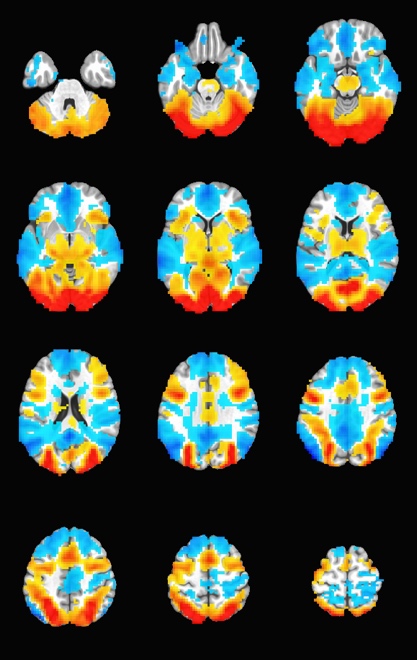

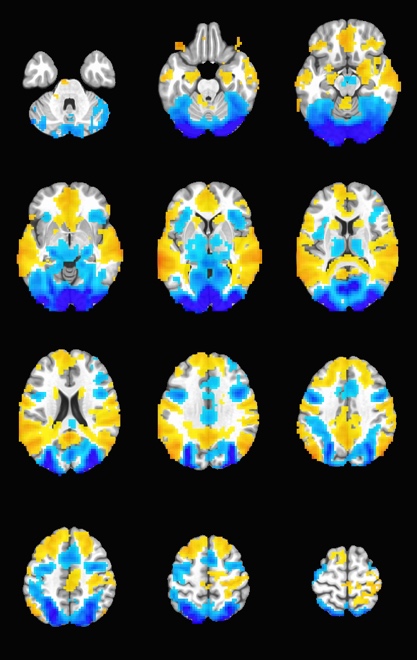


**Figure S4. Task-related activation.** Activation is shown related to the 2-back task (A), the mSDMT (B) and the difference between the two (C). For (A) and (B), warm colors represent areas positively related to the task and cool colors represent areas negatively related to the task. For (C), warm colors represent areas where the 2-back task was more active than the mSDMT and cool colors represent the reverse. For all images, the right is shown on the right and activation is represented in percent signal change.

**Brain areas showing a difference between the 2-back task and the mSDMT**

| **Location** | **Direction** | **X** | **Y** | **Z** | **Vox** | **Z Stat** |
| --- | --- | --- | --- | --- | --- | --- |
| Middle Frontal Gyrus | mSDMT > 2-back | 38.1 | 50.2 | 30 | 43 | -4.99 |
| Inferior Frontal Gyrus | 2-back > mSDMT | 41.5 | 43.3 | -22 | 75 | 3.09 |
| Inferior Frontal Gyrus | 2-back > mSDMT | 55.3 | 33.0 | -10 | 52 | 3.67 |
| Supplementary Motor Area | mSDMT > 2-back | 3.7 | 9.0 | 54 | 511 | -11.45 |
| Inferior Frontal Gyrus | mSDMT > 2-back | 41.5 | 9.0 | 30 | 671 | -11.97 |
| Middle Temporal Gyrus | 2-back > mSDMT | -68.5 | -42.6 | 6 | 8064 | 11.50 |
| Calcarine Gyrus | mSDMT > 2-back | 0.3 | -87.3 | -6 | 8063 | -23.09 |
| Cerebellum IV-V | 2-back > mSDMT | -20.4 | -35.7 | -34 | 85 | 3.46 |
| **Table S2. The brain areas showing differential activation between the tasks.** X Y Z = the location of the voxel with peak intensity in each cluster; Vox refers to the number of voxels in the cluster; Z Stat refers to the maximal Z statistic in each cluster. The sign of the Z statistic and the Direction column show which task was more active (positive for the 2-back and negative for the mSDMT). | | | | | | |

**References**

1. Avants BB, Epstein CL, Grossman M, Gee JC (2008) Symmetric diffeomorphic image registration with cross-correlation: Evaluating automated labeling of elderly and neurodegenerative brain. Med Image Anal 12:26–41. doi: 10.1016/j.media.2007.06.004

2. Behzadi Y, Restom K, Liau J, Liu TT (2007) A component based noise correction method (CompCor) for BOLD and perfusion based fMRI. Neuroimage 37:90–101. doi: 10.1016/j.neuroimage.2007.04.042

3. Cox RW, Hyde JS Software tools for analysis and visualization of fMRI data. NMR Biomed 10:171–8

4. Fonov V, Evans A, McKinstry R, Almli C, Collins D (2009) Unbiased nonlinear average age-appropriate brain templates from birth to adulthood. Neuroimage 47:S102. doi: 10.1016/s1053-8119(09)70884-5

5. Greve DN, Fischl B (2009) Accurate and robust brain image alignment using boundary-based registration. Neuroimage 48:63–72. doi: 10.1016/j.neuroimage.2009.06.060

6. Jenkinson M, Bannister P, Brady M, Smith S (2002) Improved optimization for the robust and accurate linear registration and motion correction of brain images. Neuroimage 17:825–41

7. Jenkinson M, Smith S (2001) Med Image Anal 2001 Jenkinson. Med Image Anal 5:143–156. doi: 10.1016/S1361-8415(01)00036-6

8. Lanczos C (1964) Evaluation of Noisy Data. J Soc Ind Appl Math Ser B Numer Anal 1:76–85. doi: 10.1137/0701007

9. Power JD, Barnes KA, Snyder AZ, Schlaggar BL, Petersen SE (2012) Spurious but systematic correlations in functional connectivity MRI networks arise from subject motion. Neuroimage 59:2142–2154. doi: 10.1016/j.neuroimage.2011.10.018

10. Power JD, Mitra A, Laumann TO, Snyder AZ, Schlaggar BL, Petersen SE (2014) Methods to detect, characterize, and remove motion artifact in resting state fMRI. Neuroimage 84:320–341. doi: 10.1016/j.neuroimage.2013.08.048

11. Satterthwaite TD, Elliott MA, Gerraty RT, Ruparel K, Loughead J, Calkins ME, Eickhoff SB, Hakonarson H, Gur RC, Gur RE, Wolf DH (2013) An improved framework for confound regression and filtering for control of motion artifact in the preprocessing of resting-state functional connectivity data. Neuroimage 64:240–256. doi: 10.1016/j.neuroimage.2012.08.052

12. Tustison NJ, Avants BB, Cook PA, Zheng Y, Egan A, Yushkevich PA, Gee JC (2010) N4ITK: Improved N3 bias correction. IEEE Trans Med Imaging 29:1310–1320. doi: 10.1109/TMI.2010.2046908

13. Zhang Y, Brady M, Smith S (2001) <00906424.Pdf>. 20:45–57. doi: 10.1016/j.bmcl.2016.12.081
